# Genome sequence of *Bradyrhizobium* sp. Ash2021, a free-living *Bradyrhizobium* species isolated from *Pinus radiata* forest soil

**DOI:** 10.1128/mra.00852-24

**Published:** 2024-12-16

**Authors:** Charlotte Armstrong, Steve Wakelin

**Affiliations:** 1 Ecology & Environment, Scion, Christchurch, New Zealand; California State University San Marcos, San Marcos, California, USA

**Keywords:** *Bradyrhizobium*, genome analysis, soil microbiology, forest soils

## Abstract

*Bradyrhizobium* sp. Ash2021 is a free-living soil bacterium isolated from a *Pinus radiata* forest in Canterbury, New Zealand. The genome comprises of a 9,328,819 bp chromosome and a 375,468 bp plasmid. The chromosome harbors biosynthesis pathways for auxins, thiazole-oxazole-modified microcins, and polyketide synthase. The plasmid harbors lactate and GlcNAc metabolism genes.

## ANNOUNCEMENT

Bradyrhizobia are the most prevalent and abundant genus of bacteria in coniferous forest soils, irrespective of location and soil type (1–3). Bradyrhizobia are known endophytes to leguminous plants due to their ability to form nodules and fix N_2_; however, recent research indicates that some Bradyrhizobia can also exist in various other ecotypes, including free-living non-symbiotic taxa (3). Except for a few strains, there is very little genomic information for free-living Bradyrhizobia, presenting a major gap in understanding one of the most abundant and widely distributed bacterial genera in forest soils globally (3).

*Bradyrhizobium* sp. Ash2021 bacteria were isolated into a pure culture from the topsoil of a *Pinus radiata* plantation forest in New Zealand (GPS: 43.18 S, 172.56 E; WGS 84) in July 2021. The soil is classified as Immature Pallic in the New Zealand Classification System (4). A soil solution was made by mixing 1 g of soil in 100 mL of sterilized ddH_2_O for 4 hours; this solution was then serially diluted to 10^−8^ g soil^−1^ mL in a biological safety cabinet. Aliquots of 100 µL were spread on Reasoner’s 2A agar, and the plates were kept at room temperature in the dark. Single colonies were selected after ~1 month incubation for analysis.

*Bradyrhizobium* sp. Ash2021 was sequenced at SeqCentre (Pittsburgh, USA). Genomic DNA was extracted using Qiagen Blood and Tissue kits per the manufacturer’s recommendations. Libraries were prepared by tagmentation using the Illumina DNA prep kit where Illumina adaptors and barcodes were added to TruSeq primers S501-S508 and N701-N712 (5). Sequencing was performed on Illumina NextSeq 550 platform with 2 × 150 bp read length. Supplementary long read sequencing was performed on Oxford Nanopore Technology (ONT) MinION R9 flow cells (R9.4.1). DNA was prepared using ONT’s ligation sequencing gDNA kit (SQK-LSK109), and no shearing or size selection was performed. Basecalling was conducted using Guppy version 5.0.16 at a high-accuracy rate. The Illumina sequencing generated 2.3 million paired-end reads, and the ONT sequencing generated 131,894 reads, with an average length of 2,441 bp, an *N*_50_ of 5,909 bp, and a maximum length of 382,303 bp.

Quality control and adapter trimming were performed with bcl2fastq version 2.20.0.445 (6) for Illumina and Porechop version 0.2.3_seqan2.1.1 (7) for ONT sequencing. Hybrid assembly with Illumina and ONT reads was performed with Unicycler version 0.4.8 with default parameters (8), and the quality of assembly was checked with QUAST version 5.0.2 (9). A complete chromosome and a single plasmid were assembled; these are summarized in Table 1. The assignment of strain Ash2021 to *Bradyrhizobium* was based on whole genome phylogeny using Genome Database Taxonomy (10).

**TABLE 1:** Assembled genome features of *Bradyrhizobium* sp. Ash2021

Contig	Size (bp)	*N*_50_ (bp)	GC%	Coverage (×)	Protein coding genes	tRNA, rRNA
Chromosome	9,328,819	9,328,819	62	71.0	9,292	52, 3
Plasmid	375,468	375,468	60	71.0	541	1, 0

Genome annotation was performed with Prokka version 1.14.5 (11), and the NCBI Prokaryotic Genome Annotation Pipeline version 6.2 (12). The chromosome includes genes predicted for denitrification with nitrite reductase and nitrous oxide reductase gene clusters. The bacterium lacks evidence of genes used for nitrogen fixation (*nif*) and nodulation (*nod*) that are normally associated with legume nodule-forming symbiotic rhizobia. This indicates that this is a non-symbiotic ecotype of *Bradyrhizobium*.

Sixteen secondary metabolite clusters were predicted with antiSMASH version 7.1.0 (13) and RAST (14), including biosynthesis genes for antimicrobial compounds, polyketide synthase genes (15), thiazole-oxazole-modified microcins (16), terpene synthesis and homoserine lactone clusters, and auxin synthesis genes.

*Bradyrhizobium* is usually considered to be unireplicon, with a few strains containing plasmids (17). Annotation of the plasmid revealed plasmid replication modules repA, B, and C, toxin-antitoxin replicon stabilization systems, N-acetylglucosamine (GlcNAc), and L-lactate utilization.

## Data Availability

The whole-genome sequencing project has been deposited at GeneBank under the accession number CP100604.1 for the chromosome and CP100605.1 for the plasmid. The Illumina and Nanopore raw reads can be accessed under the SRA accession numbers SRX25634638 and SRX25634639, respectively. All annotations generated by antiSMASH and RAST are publicly available on Figshare (https://doi.org/10.6084/m9.figshare.27610341.v1).
